# Screen Time, Age and Sunshine Duration Rather Than Outdoor Activity Time Are Related to Nutritional Vitamin D Status in Children With ASD

**DOI:** 10.3389/fped.2021.806981

**Published:** 2022-01-13

**Authors:** Ling Shan, Hanyu Dong, Tiantian Wang, Junyan Feng, Feiyong Jia

**Affiliations:** Department of Developmental and Behavioral Pediatrics, The First Hospital of Jilin University, Changchun, China

**Keywords:** autism spectrum disorder, sedentary behavior, multiple linear regression, environmental factor, 25(OH)D

## Abstract

**Objective:** This study aimed to investigate the possible association among vitamin D, screen time and other factors that might affect the concentration of vitamin D in children with autism spectrum disorder (ASD).

**Methods:** In total, 306 children with ASD were recruited, and data, including their age, sex, height, weight, screen time, time of outdoor activity, ASD symptoms [including Autism Behavior Checklist (ABC), Childhood Autism Rating Scale (CARS) and Autism Diagnostic Observation Schedule–Second Edition (ADOS-2)] and vitamin D concentrations, were collected. A multiple linear regression model was used to analyze the factors related to the vitamin D concentration.

**Results:** A multiple linear regression analysis showed that screen time (β = −0.122, *P* = 0.032), age (β = −0.233, *P* < 0.001), and blood collection month (reflecting sunshine duration) (β = 0.177, *P* = 0.004) were statistically significant. The vitamin D concentration in the children with ASD was negatively correlated with screen time and age and positively correlated with sunshine duration.

**Conclusion:** The vitamin D levels in children with ASD are related to electronic screen time, age and sunshine duration. Since age and season are uncontrollable, identifying the length of screen time in children with ASD could provide a basis for the clinical management of their vitamin D nutritional status.

## Introduction

Autism spectrum disorder (ASD) is characterized in the Diagnostic and Statistical Manual of Mental Disorders-fifth edition (DSM-5) by persistent deficits in social interaction and communication and stereotyped or repetitive patterns of behavior, interests or activities (1). The latest ASD prevalence (2) reported by the U.S. Centers for Disease Control and Prevention (CDC) in 2020 was one in 54 children at the age of 8 years. Currently, ASD is a relatively common neurodevelopmental disorder in children that has a serious impact on children's social adaptability. Unfortunately, the etiology of ASD is unclear. Recent research (3, 4) has shown that ASD is the result of a combination of genetic and environmental factors. Environmental factors could include nutritional factors, heavy metal exposure, air pollution, socioeconomic factors (including lifestyles), etc. Vitamin D might be an environmental factor involved in ASD (5, 6), and screen time has been proven to influence childhood development and social behaviors (7, 8).

Previous studies (9–11) have shown that the vitamin D levels in children with ASD are lower than those in typically developing children. Furthermore, there are negative correlations between the vitamin D levels and core symptoms of ASD (12). Moreover, vitamin D supplementation might improve the core symptoms of ASD (5, 11, 13). Skin under sun irradiation is a major source of vitamin D *in vivo*. Other factors (14) also affect the vitamin D concentrations, including genetic polymorphisms, age, geographical location and latitude, lifestyle (exposure behavior and culture), UVB dose, clothing and body surface area (BSA) exposure.

A sedentary lifestyle (15, 16) is an important cause of an insufficient vitamin D status. Zittermann (15) reported that adult male subjects with low levels of physical activity have lower blood vitamin D concentrations. Solis-Urra's study (16) showed that greater sedentary time is associated with vitamin D deficiency in adult and older women. Some studies have distinguished among various types of sedentary behavior (17, 18). Social activities, such as talking or hanging around, reading and playing musical instruments, are regarded as nonscreen-based sedentary behavior, whereas watching television (TV) and videos and playing traditional video games are regarded as screen-based sedentary behavior. Therefore, the use of electronic devices is an important aspect of sedentary behavior. Children with ASD could have longer screen times (19, 20). The latest World Health Organization (WHO) Guidelines (21) on Physical Activity and Sedentary Behavior released in 2020 suggested that children and adolescents should limit the amount of time spent being sedentary, particularly the amount of recreational screen time.

There have been limited studies concerning vitamin D levels and children's screen time. Soden and coworkers (22) showed that ~54% of ASD children had insufficient serum 25-hydroxyvitamin D levels, and the mean electronic media use was 251 min/day; however, these authors did not consider the association between these two factors. Absoud's study (23) showed that vitamin D deficiency occurs in children (not ASD children) who exercised less outdoors, watched more TV, and were overweight. To date, no studies considered the relationship between the vitamin D levels and electronic screen time of ASD children. Based on the above studies, we hypothesize that the excessive screen time of children with ASD could be related to insufficient vitamin D concentrations due to decreased sun exposure because of less outdoor activity.

Our team conducted several preliminary studies investigating the relationship among ASD, vitamin D (5, 9, 12) and the screen activities of children with ASD (7, 8). Based on previous research, we conducted this study to explore the associations among vitamin D, screen time and other factors that can affect the concentration of vitamin D in children with ASD, such as age, sunshine duration, Body Mass Index (BMI), and outdoor activity. This study aimed to further reveal the environmental factors of ASD and provide evidence for the clinical management of vitamin D levels in children with ASD.

## Methods

### Participants

In total, 306 children diagnosed with ASD for the first time in the Department of Developmental and Behavioral Pediatrics of the First Hospital of Jilin University were recruited for this study. Recruitment started in March 2021 and was completed in August 2021. Inclusion criteria are as following. All children were from northeastern China (38°N−53°N) with an age under 7 years-old. The DSM-5 and Autism Diagnostic Observation Schedule–Second Edition (ADOS-2) were utilized for the diagnosis of ASD. The participants were diagnosed for the first time and without systematic intervention. The Autism Behavior Checklist (ABC) and Childhood Autism Rating Scale (CARS) were also used to evaluate the symptoms of ASD to assist in the diagnosis of ASD. Exclusion criteria are: children with severe physical disabilities, uncontrolled epilepsy, vitamin D supplementation for the past 3 months, and clear metabolic diseases or genetic diseases. The study was approved by the ethics committee of our hospital, and informed consent was provided by the parents or caregivers of the children.

### Procedures

We investigated the children's characteristics (age, sex, height, and weight), ASD symptoms, outdoor activity time, screen time and serum concentration of vitamin D. Height and weight were measured by physicians in the clinic. The parents provided the children's other basic characteristics and mean outdoor activity time per day when visiting the evaluator. The children's ASD symptoms were examined using the ABC and CARS. The ABC is a 57-item screening checklist for autistic symptoms containing five subscales (body behavior, sensory, self-care, language and social interaction). This scale is designed for parent interviews. The CARS consists of 15 subscales, each of which is scored on a continuum from normal to severely abnormal. The CARS requires observation of the behavior of ASD children in a consulting room. The CARS was evaluated by experienced evaluators from our department. The evaluator also collected the screen time per day on weekdays and weekends and calculated and recorded the average daily screen time as follows: average daily screen time (hours)= [screen time per day on weekdays (min)^*^5+screen time per day on weekends (min)^*^2]/7/60. The ADOS-2 was utilized in this study as a diagnostic tool for ASD. The ADOS-2 (24) is a semistructured, standardized assessment tool for individuals with suspected ASD and measures autism symptoms in the domains of social relatedness, communication, play, and repetitive behaviors; the ADOS-2 is considered the gold standard for ASD diagnostic evaluation. We also tested the serum vitamin D concentration of the children with ASD. 25-Hydroxyvitamin D (25(OH)D) is the main circulating form of vitamin D. Therefore, we measured the concentration of 25(OH)D to reflect the nutritional status of vitamin D in the children with ASD. All samples were tested by Guangzhou KingMed Diagnostics Group Co., Ltd. (KingMed Diagnostics, SSE 603882) using the liquid chromatography tandem mass spectrometry method.

### Statistical Analysis

We used Statistical Product and Service Solutions (SPSS) software version 23.0 (SPSS for Windows, SPSS Inc., Chicago, IL, USA) to analyze all data. The continuous variables with normal distributions are represented as the means ± standard deviations (SDs), and the categorical variables are represented as frequencies (percentages). The continuous variables with normal distributions were compared by Student's *t*-test or ANOVA. The correlations among the serum concentration of vitamin D, age, and screen time were detected by a Pearson's correlation test. A multiple linear regression model was used to analyze the factors related to the vitamin D concentrations. The results were considered significant at *P* < 0.05.

## Results

**Figure 1:**
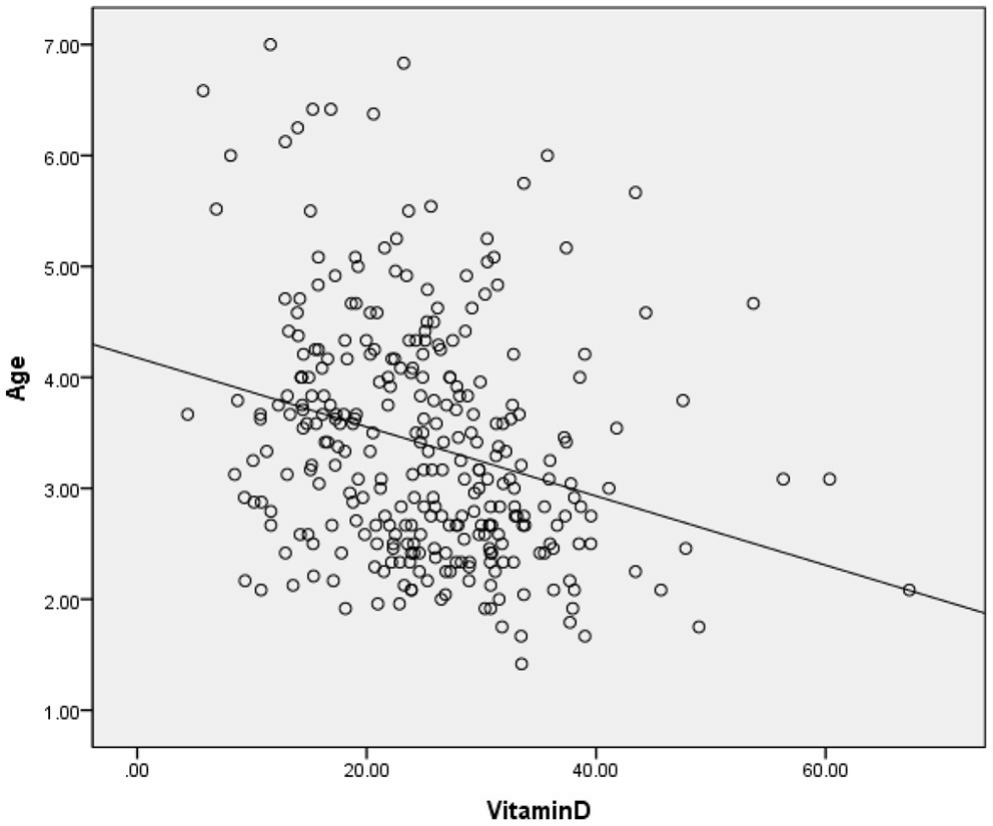
Correlation of vitamin D (ng/ml) and age (y).

**Figure 2:**
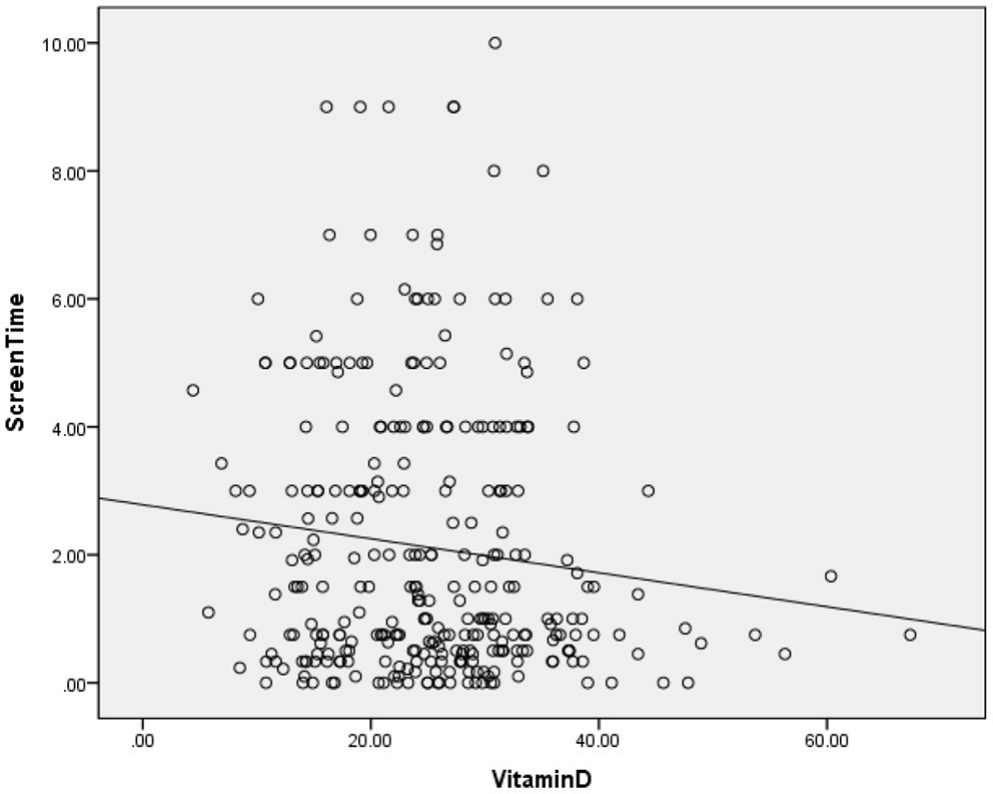
Correlation of vitamin D (ng/ml) and screen time (h).

The clinical and sociodemographic characteristics are presented in Table 1. There were 233 boys and 73 girls among the children with ASD (76.14 vs. 23.86%). Their age ranged from 1.7 to 7 years old (3.39 ± 1.07 y). Their mean screen time was 2.12 ± 2.14 h per day. The mean concentration of serum 25(OH)D was 25.26 ± 9.29 ng/ml. We grouped the ASD children according to sex, BMI, time of outdoor activities and blood collection month (reflecting the sunshine duration) and compared the vitamin D concentrations among the groups (Table 2). The vitamin D concentration was not statistically significant in the comparison between the male and female groups (*t* = −0.537, *P* = 0.591). We calculated the BMI of all enrolled children (kg/m^2^). According to their BMI (25), the children with ASD were divided into normal or underweight, overweight and obese groups. The comparison of the vitamin D levels among the groups was not statistically significant (*F* = 1.441, *P* = 0.239). However, as the BMI increased, the vitamin D levels tended to decrease (Table 2). According to the time of outdoor activities per day, we divided the children into four groups (<30 min, ≥30 and <60 min, ≥60 and <90 min, ≥90 min). The comparison of the vitamin D levels among the groups was not statistically significant (*F* = 1.193, *P* = 0.313). However, as the outdoor activity time increased, the vitamin D levels tended to increase (Table 2). According to the blood collection month, we divided the children into six groups (March, April, May, June, July, and August). The comparison of the vitamin D levels among the groups was statistically significant (*F* = 2.728, *P* = 0.020). It seems that the longer the sunshine duration, the higher the vitamin D concentration (Table 2). The correlation analysis showed that the vitamin D concentration of the children with ASD was negatively correlated with age (*r* = −0.115, *P* = 0.045) and screen time (*r* = −0.272, *P* < 0.001) (Table 3; Figures 1, 2). The older the age and the longer the screen time, the lower the vitamin D concentration.

**Table 1:** Patient characteristics.

	***N* = 306**
Age (M ± SD) (years)	3.39 ± 1.07
Vitamin D (M ± SD) (ng/ml)	25.26 ± 9.29
Screen time (M ± SD) (hours)	2.12 ± 2.14
ABC^a^ score (M ± SD)	53.31 ± 16.31
CARS^b^ score (M ± SD)	33.92 ± 4.36

a *ABC, Autism Behavior Checklist*.

b *CARS, Childhood Autism Rating Scale*.

**Table 2:** Comparison of vitamin D in each group (grouped by gender, BMI, time of outdoor activities and blood collection month).

	***N* (%)**	**Vitamin D** **(M ± SD)** **(ng/ml)**	**t/F**	***P***
Gender			−0.537	0.591
Male	233 (76.1)	25.10 ± 9.05		
Female	73 (23.9)	25.77 ± 10.04		
BMI^a^			1.441	0.239
Normal or underweight	177 (61.0)	25.87 ± 9.48		
Overweight	61 (21.0)	25.30 ± 10.12		
Obese	52 (18.0)	23.37 ± 7.53		
Time of outdoor activities^b^			1.193	0.313
<30 min	89 (29.6)	24.20 ± 10.14		
≥30 and <60 min,	94 (31.2)	25.00 ± 8.76		
≥60 and <90 min	53 (17.6)	25.81 ± 8.15		
≥90 min	65 (21.6)	26.96 ± 9.77		
Blood collection month			2.728	0.020^*^
March	100 (32.7)	22.86 ± 8.98		
April	80 (26.1)	24.98 ± 11.38		
May	36 (11.8)	26.89 ± 9.61		
June	46 (15.0)	27.24 ± 7.67		
July	26 (8.5)	27.95 ± 4.87		
August	18 (5.9)	27.64 ± 5.36		

a *Body Mass Index (BMI) data were not available for 16 children*.

b *Time of outdoor activities data were not available for five children*.

* *P < 0.05*.

**Table 3:** Correlations among vitamin D, age, and screen time.

	***R***	***P***
Age	−0.115	0.045^*^
Screen time	−0.272	<0.001^*^

* *P < 0.05*.

We incorporated age, screen time, BMI, time of outdoor activity, and blood collection month into a multiple linear regression model (Table 4) with vitamin D as the dependent variable. We analyzed whether these factors were related to the vitamin D concentrations in the children with ASD. The results of the multiple linear regression showed that age (β = −0.233, *P* < 0.001), screen time (β = −0.122, *P* = 0.032) and blood collection month (β = 0.177, *P* = 0.004) were related to the vitamin D concentrations in the children with ASD.

**Table 4:** Multiple linear regression model of vitamin D.

	**β**	**T**	**95% CI**	**P**
Age	−0.233	−4.076	−0.260, −0.091	<0.001^*^
Screen time	−0.122	−2.157	−0.017, −0.001	0.032^*^
Body Mass Index (BMI)	−0.059	−1.038	−2.071, 0.641	0.300
Time of outdoor activities	0.014	0.235	−0.887, 1.127	0.814
Blood collection month	0.177	2.928	0.346, 1.767	0.004^*^

* *P < 0.05*.

## Discussion

Our results suggest that the vitamin D concentrations in children with ASD are negatively correlated with screen time, and other factors that might be related to the vitamin D concentration include age and sunshine duration.

### Screen Time and Vitamin D

Our results suggest that there is an association between screen time and the vitamin D concentrations in children with ASD. A study (26) based on adults suggested that screen time could be related to a lack of time for physical activity. A Brazilian study (27) involving 12- to 17-year-old adolescents also showed that they spend a significant amount of time each day in front of electronic screens, while ~50% of teenagers do not engage in any physical activity in their spare time. Dong (28) conducted a study involving 559 adolescents aged 14 to 18 years in the southern USA and identified physical activity to be associated with the plasma 25(OH)D concentrations. Lenders (29) also reached a similar conclusion that physical activity was positively associated with the 25(OH)D levels, although the sample size was small. As mentioned earlier, we speculate that a very long screen time (as one of the most important sedentary behaviors of children) might affect children's outdoor activity time and further affect their vitamin D levels.

However, our results do not seem to fully support this speculation. Our results suggest that screen time is related to the vitamin D concentrations, but the outdoor activity time is not related to the vitamin D concentrations. Although our data show that vitamin D has a tendency to increase with increasing outdoor activity time, it is not statistically significant. According to a 2014 meta-analysis (30), sedentary behavior and physical activity were negatively correlated in young people, but the effect size was small, indicating that longer sedentary behavior cannot be completely equal to shorter activity times. Thus, sedentary behavior and less physical activity are different behaviors (31, 32), and their effects on the vitamin D concentrations cannot be substituted for each other. Inactivity and screen time might have distinct pathophysiological mechanisms and implications for illness (33). Our results suggest that screen time (but not outdoor time) is associated with the vitamin D levels, which is not ambivalent.

Another study showed a different result. A 2019 Brazilian study showed that moderate-to-vigorous physical activity could play an important role in increasing serum 25(OH)D concentrations in adolescence, especially in boys, regardless of the screen time. The sample size of the study was large (*n* = 1,152), but the subjects were typically developing adolescents aged 12–17 years, who were much older than our participants. Since vitamin D in children with ASD might have different metabolic statuses (6) and related metabolic gene polymorphisms (34, 35) compared with typically developing children, the associations among screen time, sedentary behavior, outdoor activity time and vitamin D deserve further discussion.

### Age and Vitamin D

Our results suggest that the age of children with ASD is negatively correlated with the vitamin D concentrations. Older children with ASD have lower vitamin D concentrations. Andiran's research (36) conducted correlation analyses in Turkey and revealed that the 25(OH)D levels were negatively correlated with age (0–5 age group, 34.2 ± 16.2 ng/ml; 5–10 age group, 20.5 ± 8.7 ng/ml; 10–16 age group, 18.7 ± 11.5 ng/ml). This finding is likely related to the preventive measures in primary health care implemented by the Ministry of Health in Turkey (36). Since 2005, vitamin D supplements have been distributed to all newborns throughout their infancy at no financial cost (37). A US survey (38) involving 4,558 children and adolescents aged 1–11 years also showed that the vitamin D concentration of the children aged 1–5 was higher than that of the children aged 6–1 (70 vs. 66 nmol/L). However, the United States is a multiracial country, and non-Hispanic black and Hispanic children have the lowest levels of 25(OH)D, which might have had an impact on the results.

The situation is different in China. Vitamin D is recommended for routine supplementation of 400–800 units from birth to early childhood (rather than school age), and it is not a free-cost drug in China. Universal primary health care for children must be further strengthened. Similarly, in the UK, a population-based study conducted in 2011 (23) also showed that the plasma vitamin D levels decreased progressively with age. Although there are no recommendations for vitamin D supplementation in older children, in younger children, the recommended supplement uptake is low. The participants in this study were all children who had not taken vitamin D regularly in the previous 3 months; thus, vitamin D supplements can be ignored. The reason for this phenomenon is not yet clear.

### Sunshine Duration and Vitamin D

Our research suggests that the vitamin D levels in the summer (June-August) are higher than those in the spring (March-May). Most humans depend on sunlight exposure to satisfy their requirements for vitamin D (39). Solar ultraviolet B photons are absorbed by the skin, leading to the transformation of 7-dehydrocholesterol into vitamin D3 (cholecalciferol) (40). Vitamin D levels are related to ultraviolet light, which is easy to understand. Similarly, some studies (41–43) have shown that season is an important factor affecting the vitamin D status. The vitamin D levels in the human body are the highest in the summer (41).

Interestingly, regarding sunshine duration, June is the longest month of sunshine in the year, but the level of vitamin D in July and August is slightly higher than that in June, which seems to be a delayed phenomenon. There was indeed a Brazilian study that supports our speculation. In Brazil, the results of the São Paulo vitamin D evaluation study (44) showed that the lowest UV radiation levels were recorded in the winter, while the lowest 25(OH)D concentrations occurred in the spring, corresponding to a delay of a season. A strong correlation was observed between the current mean 25(OH)D concentration and the mean UVR value from the previous season (*r* = 0.98) (45).

### Possible Intervention Strategies

We recommend limiting the screen time of children with ASD. Children younger than 2 years who have a deviation in social and language development but have not yet been confirmed with a diagnosis of ASD according to the AAP recommendation (46) should avoid electronic screen devices. The limited screen time requires high-quality content, high-quality company and interaction with parents. In addition, it is still recommended that children with ASD take vitamin D while monitoring their vitamin D levels, especially those who are older and have longer screen times, during short sunshine duration seasons and in areas with high latitudes (low UV), emphasizing the management of multilevel related environmental factors, in addition to behavioral interventions and education for children with ASD. In the future, a cohort study will be performed to verify the effectiveness of our management strategy.

### Limitations and Further Directions

We ignored the vitamin D intake in the diet. Although vitamin D produced through the skin is the most important source, under the condition of insufficient sunlight (especially in the spring at high latitudes in northeastern China), food can supply ~10 to 20% of vitamin D (39). Future research should consider dietary factors.

This study is only a cross-sectional study and cannot provide causal conclusions. Further prospective cohort studies are needed.

We only investigated the vitamin D levels of children with ASD in the spring and summer, while the sunshine duration is shorter in the autumn and winter. Therefore, we must conduct a whole year of research in the future to verify our conclusions.

## Conclusion

The vitamin D levels in children with ASD are related to their electronic screen time, age and sunshine duration. While age and season are uncontrollable, identifying the length of screen time in children with ASD could provide a basis for the clinical management of their vitamin D levels.

## Data Availability Statement

The raw data supporting the conclusions of this article will be made available by the authors, without undue reservation.

## Ethics Statement

The studies involving human participants were reviewed and approved by the Ethics Committee of the First Hospital of Jilin University. Written informed consent to participate in this study was provided by the participants' legal guardian/next of kin.

## Author Contributions

LS: methodology, investigation, and writing the initial manuscript. HD: methodology, investigation, formal analysis, and some writing. TW: data curation and formal analysis and editing the manuscript. JF: investigation, formal analysis, and editing the manuscript. FJ: conceptualization, funding acquisition, supervision, and oversight and resources. All authors contributed to the article and approved the submitted version.

## Funding

This work was supported by the National Natural Science Foundation of China (Grant Number: 81973054), Key Scientific and Technological Projects of Guangdong Province (Grant Number: 2018B030335001), Joint Fund Bethune Medical Special Project of Jilin Province (Grant Number: 20200201507JC), and the Project of Jilin Provincial Department of Finance (Grant Number: 2018SCZWSZX-60).

## Conflict of Interest

The authors declare that the research was conducted in the absence of any commercial or financial relationships that could be construed as a potential conflict of interest.

## Publisher's Note

All claims expressed in this article are solely those of the authors and do not necessarily represent those of their affiliated organizations, or those of the publisher, the editors and the reviewers. Any product that may be evaluated in this article, or claim that may be made by its manufacturer, is not guaranteed or endorsed by the publisher.
